# Genetic impact of *Rht* dwarfing genes on grain micronutrients concentration in wheat

**DOI:** 10.1016/j.fcr.2017.09.030

**Published:** 2017-12

**Authors:** Govindan Velu, Ravi P. Singh, Julio Huerta, Carlos Guzmán

**Affiliations:** a Global Wheat Program, International Maize and Wheat Improvement Center (CIMMYT), Apdo Postal 6-641, Mexico DF, Mexico; b Campo Experimental Valle de Mexico INIFAP, Apdo. Postal 10, 56230, Chapingo, Edo de Mexico, Mexico

**Keywords:** *Rht* dwarfing genes, Isogenic lines, Micronutrients, Biofortification, Wheat

## Abstract

Wheat is a major staple food crop providing about 20% of dietary energy and proteins, and food products made of whole grain wheat are a major source of micronutrients like Zinc (Zn), Iron (Fe), Manganese (Mn), Magnesium (Mg), Vitamin B and E. Wheat provides about 40% intake of essential micronutrients by humans in the developing countries relying on wheat based diets. Varieties with genetically enhanced levels of grain micronutrient concentrations can provide a cost-effective and sustainable option to resource poor wheat consumers. To determine the effects of commonly deployed dwarfing genes on wheat grain Zn, Fe, Mn and Mg concentrations, nine bread wheat (*Triticum aestivum*) and six durum wheat (*T. turgidum*) isoline pairs differing for *Rht1* (= *Rht-B1b*) and one bread wheat pair for *Rht2* (= *Rht-D1b*) dwarfing genes were evaluated for three crop seasons at N.E. Borlaug Research Station, Cd. Obregon, Sonora, Mexico. Presence of dwarfing genes have significantly reduced grain Zn concentration by 3.9 ppm (range 1.9-10.0 ppm), and Fe by 3.2 ppm (range 1.0-14.4 ppm). On the average, about 94 ppm Mg and 6 ppm Mn reductions occurred in semidwarf varieties compared to tall varieties. The thousand kernel weight (TKW) of semidwarf isolines was 2.6 g (range 0.7-5.6 g) lower than the tall counterparts whereas the plant height decreased by 25 cm (range 16–37 cm). Reductions for all traits in semidwarfs were genotype dependent and the magnitude of height reductions did not correlate with reductions in micronutrient concentrations in wheat grain. We conclude that increased grain yield potential of semidwarf wheat varieties is associated with reduced grain micronutrient concentrations; however, the magnitude of reductions in micronutrients varied depending on genetic background and their associated pleiotropic effect on yield components.

## Highlights

• The Rht dwarfing genes decreased micronutrient concentrations, however, the magnitude depends on the genetic background.
• There was a negative effect on kernel weight indicating that Rht genes increased the number of kernels per spike as well as kernels per unit area.
• Highly significant positive correlation between the micronutrients; rate of reductions differs in different genetic background.

## 1 Introduction

Globally, over 805 million people suffer from hunger and approximately 165 million (or 1 in 4) children under the age of five are stunted due to the lack of proper nutrition received between pregnancy and a child's second birthday (FAO, 2017). Despite the significant growth in agricultural production, a large population suffers from the dietary deficiency of essential micronutrients such as zinc (Zn) and iron (Fe). Additionally, magnesium (Mg) and manganese (Mn) deficiency is very common among resource poor consumers. In particular women and children are more vulnerable to the micronutrient deficiency. The first 1,000-days are the most important time period for a child's cognitive, intellectual and physical development. Under nutrition contributes to 45 percent of the child deaths each year worldwide (WHO, 2017). Biofortification offers a sustainable solution to increase food and nutritional security to millions of resource poor consumers depending on major staples as the main source of their dietary energy (Bouis et al., 2011). To meet the challenge of improving nutritional food security, the HarvestPlus program of the CGIAR research program on Agriculture for Nutrition and Health (CRP-A4NH) supports the development of micronutrient-rich staple crops including common wheat (*Triticum aestivum* L.). The primary target nutrient for wheat is Zn, as millions of resource poor wheat consumers in the target countries in South Asia and Africa are prone to Zn deficiency. In fact, more than 400,000 children die each year due to Zn deficiency globally (Muthayya et al., 2013). Overall, an estimated 17.3% of the global population is at risk of inadequate zinc intake. The regional estimated prevalence of inadequate Zn intake ranges from 7.5% in high-income regions to 30% in South Asia (Stein, 2010). Multiple micronutrient deficiencies, including Mg and Mn, are widespread and have severe health consequences in resource poor communities. Wheat varieties with improved nutritional quality, protein content, high grain yield and desirable processing quality in adapted genetic backgrounds can help alleviate nutrient deficiencies among resource poor people (Pfeiffer and McClafferty, 2007, Singh and Velu, 2017). For this reason, genetic resources (landraces and ancestors of common wheat) with high Zn and Fe content such as *Aegilops tauschii*, *T. turgidum* ssp. *diccocoides, T. turgidum* ssp. *dicoccum* and *T.aestivum* ssp. *spelta* species, have been used in breeding to enhance Zn concentration (Ortiz-Monasterio et al., 2007, Guzmán et al., 2014, Velu et al., 2011, Velu et al., 2012, Velu et al., 2014).

Plant height is an important trait in wheat, it significantly reduces lodging in higher yielding environments and increases grain yield. Extensive research has been conducted to study the effect of *Rht* genes on grain yield (Borlaug, 1968, Villareal and Rajaram, 1992) and to some extent on protein content (Gooding et al., 1999, McClung et al., 1986). The widely deployed dwarfing genes *Rht1* (*Rht-B1b*) and *Rht2* (*Rht-D1b*) are known to have pleiotropic effects on input responsiveness and lodging tolerance that leads to significant grain yield increases in wheat. This phenomenon led to the wide adoption of semidwarf wheat varieties in 1960′s and 1970′s resulting in Green Revolution in South Asia and other regions of the world (Swaminathan 2013).

New research was undertaken at the International Maize and Wheat Improvement Center (CIMMYT) in Mexico where the Green Revolution began using the *Rht* dwarfing genes. The objective of our study was to evaluate effects of dwarfing genes *Rht1* and *Rht2* on four essential micronutrients (Fe, Zn, Mn and Mg) concentration and associated pleiotropic effects on grain weight and plant height by using 16 pairs of isogenic lines developed previously for 10 bread wheat (*T. aestivum*) and 6 durum wheat (*T. turgidum*) varieties.

## 2 Materials and methods

### 2.1 Plant materials

Sixteen pairs of tall and semi-dwarf isolines derived from CIMMYT historic and modern wheat varieties were used in this study. Ten pairs of isolines are derived from bread wheat (*T. aestivum*) and six were from pasta wheat (*T. durum*) varieties. The detailed procedure in developing these isolines has been described in Singh et al. (2001). All isolines carry *Rht1* dwarfing gene excepting Pavon isolines, which carries *Rht2* gene.

### 2.2 Trial design and management

The sixteen pairs of isolines were grown in a split-plot design with two replicates during 2014-15 (2015), 2015-16 (2016) and 2016-17 (2017) crop seasons at Norman E. Borlaug Experimental Station in Ciudad Obregon, Sonora, Mexico. Each genotype was planted in a paired row of 1 m long with a bed to bed distance of 80 cm. Trials were laid out in a paired split-plot design with the isolines (tall and semidwarf) as main plots and genotypes as subplot factors. All recommended agronomic practices were followed (Velu et al., 2012, Velu et al., 2017). The commercial form of ZnSO4·7H_2_O was applied in the soil as basal application along with the 50% of the recommended 200 kg/ha Nitrogen and 100% of 50 kg/ha Phosphorus fertilizers. Remaining 50% or 100 kg/ha N applied as top dressing during the second irrigation about 30 days of sowing. At maturity, whole plots were harvested.

### 2.3 Micronutrient analysis

About 30 g of grain samples free from dust particles, chaff, glumes and other plant materials was prepared for determining micronutrient concentration and thousand kernel weight (TKW). Grain Fe and Zn concentrations (parts per million: ppm) were measured using a bench-top, non-destructive, energy-dispersive X-ray fluorescence spectrometry (EDXRF) instrument (model X-Supreme 8000, Oxford Instruments plc, Abingdon, UK), calibrated for high-throughput screening of Zn and Fe in whole wheat grain (Paltridge et al., 2012). In addition, grain samples were analyzed for all four micronutrients with Inductively Coupled Plasma Mass Spectroscopy (ICP-MS) at Flinders University, Australia. TKW was measured with a SeedCount digital imaging system (model SC5000, Next Instruments Pty Ltd, New South Wales, Australia). Plant height was measured from bottom of the plants to tip of the awns after physiological maturity of each plot.

### 2.4 Statistical analysis

Statistical analyses were conducted using Statistical Analysis System 9.2 (SAS Institute, Cary, NC, USA). Analysis of variance was done following fixed model (Gomez and Gomez, 1984), and data was analyzed as a paired split-plot design. Mean comparisons between tall and semi-dwarf pairs were also made for all six traits in the study. Broad-sense heritability (H^2^) (repeatability) was estimated across environments using the formula H^2^ = σg^2^/(σg^2^ + σge^2^/y + σe^2^/ry), where σg^2^ is the genotypic variance, σge^2^ is the GE variance, and σe^2^ is the residual error variance for r replicates and y years. The Principal Component Analysis (PCA) was calculated using META-R statistical package (www.data.cimmyt.org). The Pearson correlation coefficient between traits was calculated using PROC CORR procedure.

## 3 Results

Analysis of variance showed highly significant differences between genetic backgrounds (entries) for grain Zn, Fe, Mn, Mg, TKW and plant height (PH) (Table 1). Combined analysis across environments showed significant environment effect on grain Zn, Mn and Mg concentrations (P <0.001). Contrast between tall and semi-dwarf isolines was significant for Fe, Zn, Mn, Mg and PH (P < 0.001) and TKW (P < 0.05). Interaction effect of *Rht* genes on environments was significant for all traits except Fe. The broad sense heritability was high for all traits (H^2^ = 0.75 to 0.95), except intermediate heritability was observed for Fe (H^2^ = 0.52) and coefficient of variation below 10% suggested a good management of trials across years.

**Table 1:** Combined P-values from the ANOVA for grain Zn, Fe, Mg and Mn concentrations, thousand kernel weight (TKW) and plant height (PH) determined during 2015, 2016 and 2017 seasons.

Source of variation	DF	Zn	Fe	Mg	Mn	TKW	PH
		Pr > F	Pr > F	Pr > F	Pr > F	Pr > F	Pr > F
Environment	2	<0.001	0.16	<0.001	<0.001	0.12	0.17
Entry	31	<0.001	<0.001	<0.001	<0.001	<0.001	<0.001
Environment × Entry	62	<0.001	0.132	<0.001	<0.001	<0.001	1.132
Isogenic	1	<0.001	<0.001	<0.001	<0.001	0.043	<0.001
Isogenic × Environment	2	0.006	0.75	<0.001	<0.001	<0.001	<0.001
Variance							
Error (a)		4.4	0.5	740.6	0.1	0.34	12.8
Error (b)		6.4	2.6	2478.1	13.3	3.5	48.2
Heritability		0.75	0.52	0.85	0.83	0.84	0.95

### 3.1 Effect of *Rht* genes on Zn, Fe, Mn, Mg, kernel weight and plant height

**Fig. 1:**
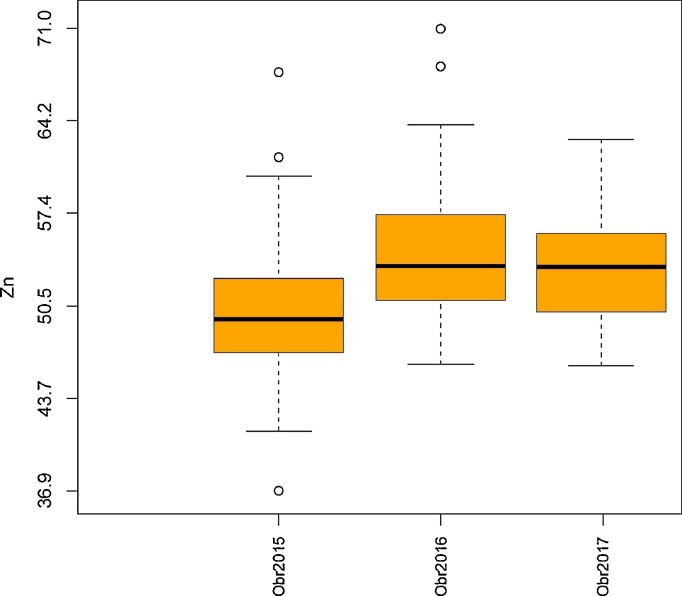
Grain Zn concentration in *Rht* isolines during 2015, 2016 and 2017.

**Fig. 2:**
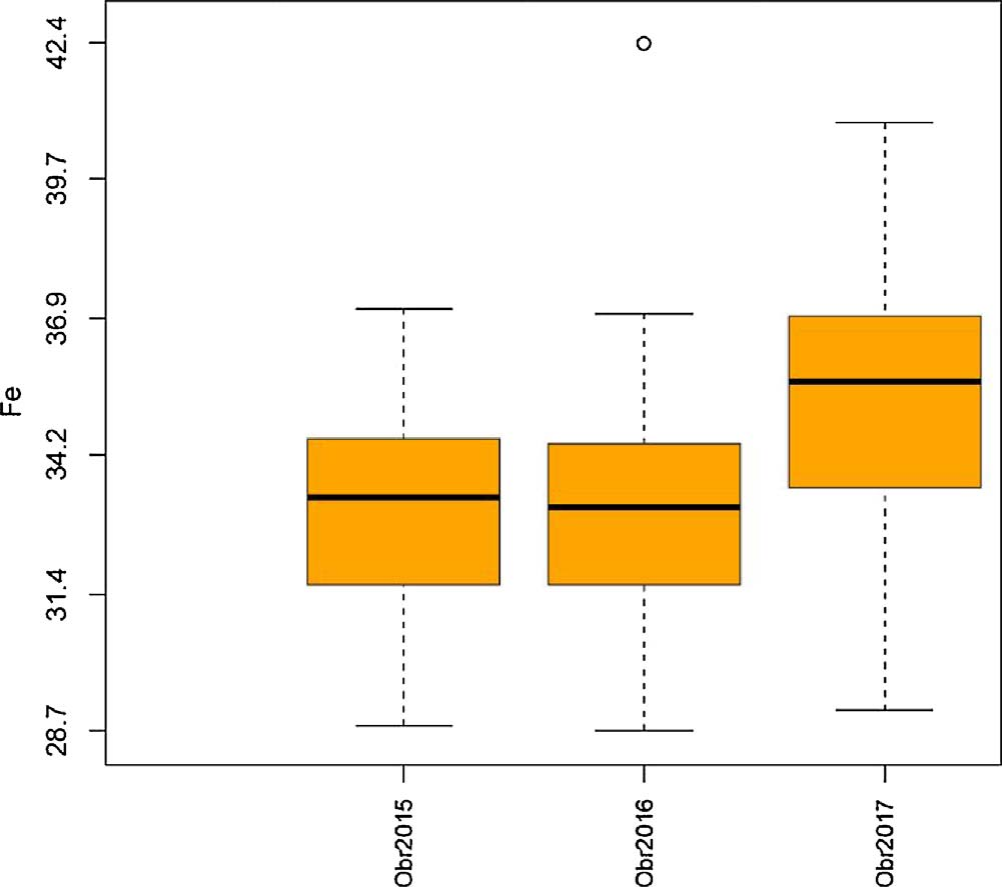
Grain Fe concentration in *Rht* isolines during 2015, 2016 and 2017.

Analysis of variance showed significant interaction effect between environments for grain Zn, Mn and Mg concentrations, however, significant positive correlations between environments allowed us to conduct combined analyses (averaged across 3 environments). Grain Zn averaged over three environments varied from 46 to 63 ppm with the mean of 52 ppm (Fig. 1), whereas grain Fe ranged from 29 to 52 ppm with the mean of 35 ppm (Fig. 2). On the average, dwarfing genes reduced grain Zn by 3.9 ppm and grain Fe by 3.2 ppm, respectively (Table 2). There was a significant genetic background effect on the expression of *Rht* genes on grain Zn and Fe concentrations, for instance highest reduction of 10 ppm occurred for Zn in bread wheat Culiacan dwarf over its tall pair, followed by durum wheat Aconchi and Bichena with about 5.9 and 5.6 ppm reductions, respectively. The lowest reduction or less effect of *Rht* dwarfing gene on grain Zn occurred in bread wheat Siete Cerros and durum wheat Focha with only 1.9 ppm reduction. In the case of Fe, the highest reduction, 14.4 ppm, occurred in bread wheat variety Genaro and the lowest in bread wheat varieties Seri with only 1 ppm difference between the isogenic pairs (Table 2). Grain Mg averaged over three environments varied from 965 to 1390 ppm with the trial mean of 1193 ppm, whereas grain Mn ranged from 40 to 64 ppm with a trial mean of 53 ppm. On average, about 94 ppm Mg and 6 ppm Mn reductions occurred in semi-dwarf varieties compared to tall varieties. The highest reduction of 150 ppm for Mg was in the wheat variety ‘Anza,’ whereas wheat variety ‘Galvez’ showed a maximum reduction of 11 ppm for Mn.

**Table 2:** Mean differences between isolines for grain Zn, Fe, Mg, Mn, TKW and PH in *Rht* isogenic lines.

Entry	Genotype	Type	Zn (ppm)	Zn_diff	Fe (ppm)	Fe_diff	Mg (ppm)	Mg_diff	Mn (ppm)	Mn_diff	TKW (g)	TKW_diff	pH (CM)	pH _diff
1	Siete cerros dwarf	BW	48.1		33.3		1155		49		42.1		91	
2	Siete cerros tall	BW	50.0	1.9	34.9	1.6	1275	120	57	8	45.7	3.7	107	17
3	Anza dwarf	BW	46.4		32.0		1240		56		37.3		88	
4	Anza tall	BW	49.9	3.5	33.5	1.6	1390	150	63	7	40.1	2.8	113	25
5	Pavon dwarf	BW	53.8		32.8		1275		53		41.5		100	
6	Pavon tall	BW	57.7	3.9	38.0	5.2	1335	60	55	2	43.6	2.1	127	27
7	Seri dwarf	BW	51.3		35.4		1215		60		42.9		92	
8	Seri tall	BW	54.0	2.7	36.4	1	1305	90	62	2	46.9	4.1	109	17
9	Kauz dwarf	BW	52.2		34.3		1075		49		40.5		91	
10	Kauz tall	BW	55.1	2.9	35.5	1.2	1095	20	50	1	43.9	3.4	110	19
11	Genaro dwarf	BW	58.9		37.6		1190		54		40.1		92	
12	Genaro tall	BW	61.6	2.7	52.0	14.4	1325	135	64	10	43.8	3.7	113	21
13	Culiacan dwarf	BW	53.0		32.0		1210		51		46.1		95	
14	Culiacan tall	BW	63.0	10	35.9	3.9	1315	105	59	7	49.0	2.8	121	26
15	Sitta dwarf	BW	49.2		31.4		1175		52		42.8		96	
16	Sitta tall	BW	51.6	2.4	34.9	3.5	1250	75	57	6	44.4	1.6	114	18
17	Nesser dwarf	BW	46.9		29.4		1120		48		36.9		83	
18	Nesser tall	BW	50.6	3.6	30.6	1.3	1215	95	52	4	42.5	5.6	107	24
19	Galvez dwarf	BW	48.5		32.8		1135		46		44.2		100	
20	Galvez tall	BW	52.8	4.3	36.1	3.3	1255	120	57	11	45.0	0.8	116	16
21	Yavaros dwarf	DW	47.5		33.6		1050		46		49.5		89	
22	Yavaros tall	DW	50.2	2.7	35.3	1.7	1135	85	52	6	53.2	3.8	122	33
23	Aconchi dwarf	DW	51.2		34.1		965		52		48.5		85	
24	Aconchi tall	DW	57.1	5.9	36.1	2	1065	100	52	0	49.5	1	121	35
25	Focha dwarf	DW	49.1		32.0		1045		42		47.6		86	
26	Focha tall	DW	51.0	1.9	35.6	3.6	1145	100	53	10	50.1	2.5	118	32
27	Lavanco dwarf	DW	49.2		33.8		1195		47		54.3		87	
28	Lavanco tall	DW	52.9	3.8	35.6	1.8	1275	80	57	10	55.0	0.7	116	29
29	Nehama dwarf	DW	49.1		33.2		1225		56		43.1		86	
30	Nehama tall	DW	54.4	5.3	36.5	3.3	1305	80	59	4	45.2	2.2	123	37
31	Bichena dwarf	DW	48.1		33.5		1015		40		46.0		98	
32	Bichena tall	DW	53.7	5.6	35.7	2.1	1080	65	41	1	47.3	1.4	120	22
Mean			52.1	3.9	34.8	3.2	1193	94	53	6	45.3	2.6	104	25
Minimum			46.4	1.9	29.4	1	965	20	40	0	36.9	0.7	83	16
Maximum			63	10	52	14.4	1390	150	64	11	55	5.6	127	37
Heritability			0.75		0.52		0.85		0.83		0.84		0.95	
LSD (5%)			4.12		7.63		116		7.3		2.12		7.8	
CV (%)			3.88		10.76		4.8		6.8		2.29		3.7	

BW: Bread Wheat, DW: Durum Wheat.

TKW = Thousand Kernel Weight (g), PH = Plant Height (cm).

A significant difference between tall and semidwarf isolines was observed for TKW with average reduction of 2.6 g for semidwarf compared to tall (Table 2). The highest and lowest reductions of 5.6 and 0.7 in TKW occurred in bread wheat Nesser and durum wheat Lavanco, respectively.

**Fig. 3:**
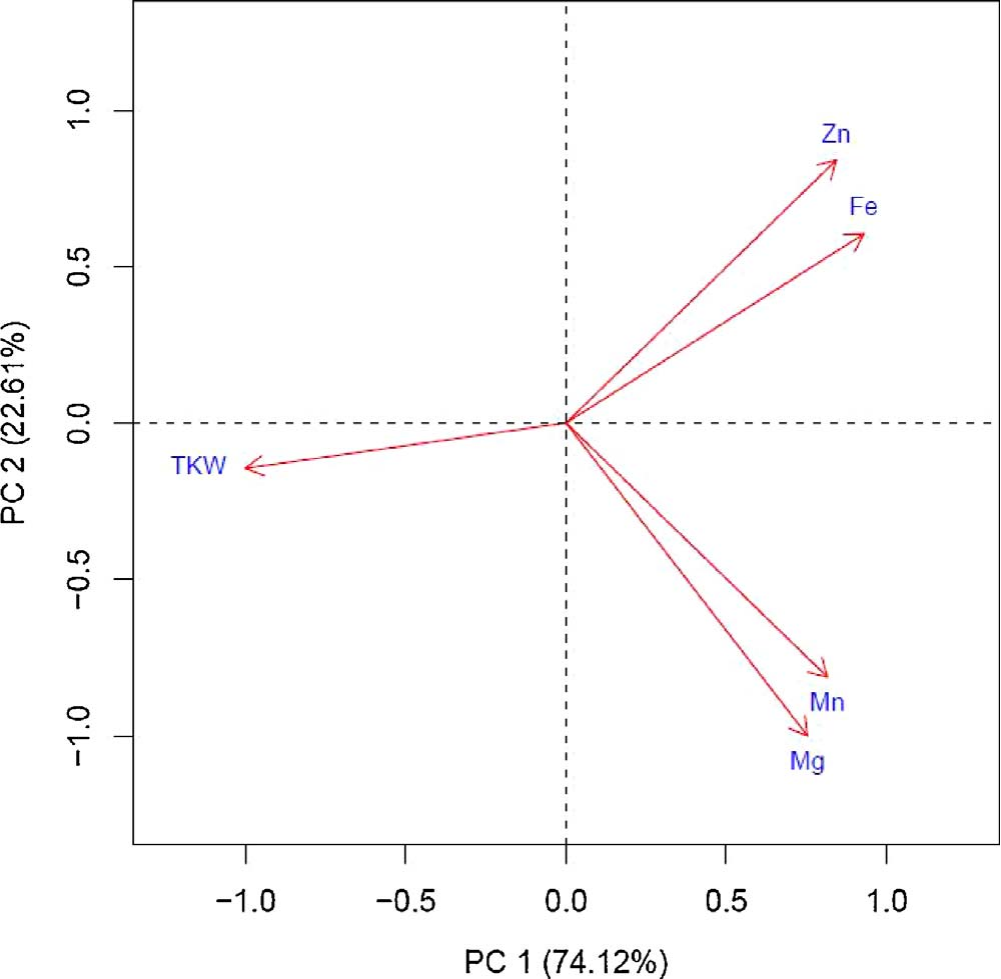
Principal component analysis (PCA) for four micronutrients and TKW in *Rht* isolines (means for 2015, 2016 and 2017).

There was a significant positive correlation between grain Zn and Fe (r = 0.45; P < 0.01) and Mg and Mn (r = 0.80; P < 0.01) and significant positive correlation between these four micronutrients. There was no correlation between these micronutrients and TKW, which was reflected from the PCA biplot (Fig. 3) where Fe and Zn were grouped together, Mg and Mn were clustered together and TKW was distantly positioned. In case of plant height, on average the semi-dwarf isolines were 25 cm shorter than the tall pairs. The lowest and highest height differences of 16 and 37 cm observed for bread wheat Galvez and durum wheat Nehama, respectively. However, magnitude of height reductions did not influence a decreased amount of Zn and Fe in semidwarfs.

## 4 Discussion

Previous studies have shown that *Rht* dwarfing genes reduced grain Zn and Fe concentrations in wheat with a limited number of isogenic pairs (Graham et al., 1999). Our results corroborates with this finding by using 16 pairs of isolines to investigate the effect of dwarfing genes on grain Zn, Fe, Mn and Mg concentrations and kernel weight in different genetic backgrounds. On an average, the reductions of 3.9, 3.2, 6.0 and 94 ppm for grain Zn, Fe, Mn and Mg, respectively were observed in semidwarf lines over their tall counterparts. However, the magnitude of reduction varied in different genetic backgrounds. Using same 16 pairs of isolines Singh et al. (2001) found significant increase for grain yield (1 t/ha) under optimally managed environment; however, the magnitude of yield increases also varied depending on the background. Several studies have been conducted to measure effect of *Rht* genes on kernel weight. In our study there was a negative effect on kernel weight suggesting that *Rht* genes might have contributed to increase the number of kernels per spike as well as kernels per unit area and thus compensated the marginal reductions in kernel weight for increased grain yield potential. Similarly, in winter wheat background *Rht* alleles have reduced grain weight and it was compensated by a 10% increase in number of grains per spike and a 13% increase in tiller numbers per square meter (Kertesz et al. (1991).

Recent QTL mapping studies at CIMMYT have identified pleiotropic QTL regions that enhance kernel size and grain Zn concentration simultaneously (Hao et al., 2014, Crespo-Herrera et al., 2016). These results indicate that Zn and Fe increase in tall varieties may be due to larger kernel size or lesser grain yield levels for tall lines. This merits further investigation.

Quantitative traits expressions are often influenced by a balance among multigenes or allelic changes at major gene loci resulting in variable expression of reductions in Zn and Fe in different genetic backgrounds. Thus, the changes in micronutrients concentrations are mainly due to the associated pleiotropic effects of dwarfing genes on increased biomass partitioning and higher harvest index. Considerably this trend led to slightly lower concentration of Zn and Fe per grain in modern wheat varieties, however, the total Zn harvested from the soil or Zn harvested per unit area is higher (data not shown). Recent QTL mapping studies in wheat have shown that there are various QTL regions for grain Zn and Fe, which are not associated with height or flowering genes (Crespo-Herrera et al., 2016, Velu et al., 2017, Krishnappa et al., 2017). These QTLs provide opportunities to select high Zn semidwarf wheat varieties for better adaptation and to achieve higher grain yield potential.

## Conflict of interest

The authors declare that they have no conflict of interest.
